# A clinical review of inhalation anesthesia with sevoflurane: from early research to emerging topics

**DOI:** 10.1007/s00540-017-2375-6

**Published:** 2017-06-05

**Authors:** Jorge D. Brioni, Shane Varughese, Raza Ahmed, Berthold Bein

**Affiliations:** 1 Global Medical Affairs, AbbVie, Inc., 1 N. Waukegan Rd., ABV1, North Chicago, IL 60064 USA; 2 Asklepios Hospital St. Georg, Lohmühlenstraße 5, D-20099 Hamburg, Germany

**Keywords:** Volatile anesthetic, Cardiac protection, Postoperative cognitive dysfunction, Emergence, Delirium

## Abstract

A large number of studies during the past two decades have demonstrated the efficacy and safety of sevoflurane across patient populations. Clinical researchers have also investigated the effects of sevoflurane, its hemodynamic characteristics, its potential protective effects on several organ systems, and the incidence of delirium and cognitive deficiency. This review examines the clinical profiles of sevoflurane and other anesthetic agents, and focuses upon emerging topics such as organ protection, postoperative cognitive deficiency and delirium, and novel ways to improve postanesthesia outcomes.

## Introduction

Sevoflurane has been in clinical use for inhalation anesthesia for more than 20 years and has been tested in numerous studies. The safety and efficacy of sevoflurane are well established and ongoing investigations have continued to more precisely define its effects in different patient populations and organ systems.

Recent studies have reported on hemodynamic and recovery characteristics following anesthesia maintenance with sevoflurane [1, 2]. A wide range of studies have suggested a cardioprotective effect of sevoflurane in cardiac surgery [3], and it may also have protective effects in other organs [4, 5]. Another topic of interest has been the potential impact of inhalational anesthesia in behavioral outcomes such as postoperative cognitive dysfunction (POCD) and postoperative delirium (POD) in pediatric, adult, and elderly patients [6–12]. This review will examine the clinical efficacy and safety of sevoflurane, and focus upon the emerging topics of organ protection, behavioral outcomes related to POCD and POD, and methods to optimize emergence from anesthesia.

## Clinical efficacy and safety of sevoflurane

### Adult patients

In clinical practice, sevoflurane and propofol are more frequently used as induction agents, as isoflurane and desflurane are more irritating to the airway [13]. However, a number of clinical studies have demonstrated that all of these drugs are efficacious and safe for induction of general anesthesia (Table 1). In one randomized, double-blind comparison of 8% sevoflurane versus propofol as induction agents, the time to anesthesia induction was longer with sevoflurane [14]. However, a study in 75 adult patients noted that the induction time was not statistically different between sevoflurane and propofol [15]. Additionally, a meta-analysis of five studies by Joo et al. concluded that time to loss of consciousness was similar for propofol and sevoflurane, with a weighted mean difference of 2.84 s (95% CI −12.36, 18.05) in favor of propofol [16].

**Table 1:** Induction of anesthesia in adult, pediatric, and elderly patients

Patient population and studies	Anesthetic agents compared	Time to induction (s ± SD)	*P* value
Adult			
Thwaites et al. [14]	SEVO (*n* = 51)	84 ± 24	<0.01
	PRO (*n* = 51)	57 ± 11	
Hall et al. [15]	SEVO in O_2_ (*n* = 25)	71 ± 37	NS
	SEVO in O_2_ and N_2_O (*n* = 25)	61 ± 24	
	PRO (*n* = 25)	60 ± 25	
Pediatric			
Lopez-Gil et al. [33]	SEVO (*n* = 60)	115 ± 67	<0.0001
	PRO (*n* = 60)	202 ± 107	
Elderly			
Yamaguchi et al. [40]	SEVO 2%/PRO (*n* = 15)	43 ± 15	<0.05
	SEVO 8% (*n* = 15)	67 ± 13	
	SEVO gradual reduction (*n* = 15)	72 ± 21	

*PRO* propofol, *SEVO* sevoflurane

Anesthesia maintenance and recovery profiles have also been investigated (Table 2). A study comparing the effectiveness of sevoflurane versus propofol in 66 patients revealed shorter times for two recovery parameters. The authors concluded that maintenance of anesthesia with sevoflurane resulted in a more favorable recovery profile, less patient movement, and more favorable hemodynamic responses [1]. In a phase 3 trial, sevoflurane was compared with isoflurane for maintenance of anesthesia. This study indicated that eye opening, response to commands, and request for first postoperative analgesia as a marker of wakefulness were more rapid following discontinuation of sevoflurane than isoflurane [17]. Several studies have compared sevoflurane with desflurane in intermediate recovery and time for discharge in ambulatory patients, and have demonstrated that emergence and/or early recovery from anesthesia are faster with desflurane than sevoflurane [2, 18–20]. This phenomenon is not unexpected due to the lower solubility of desflurane in blood and tissues [21], but the faster wake-up time has generally failed to translate into early readiness for discharge [2, 19, 20].

**Table 2:** Emergence and discharge times following anesthetic discontinuation (adult patients)

Study	Anesthetic agents compared	Extubation/LMA removal (min ± SD)	*P* value	Eye opening (min ± SD)	*P* value	Discharge (min ± SD)	*P* value
Choi et al. [1]	SEVO (*n* = 33)	8.2 ± 2.8	0.01	6.7 ± 2.9	0.002		
	PRO (*n* = 33)	10.4 ± 2.8		9.2 ± 2.6			
Campbell et al. [17]	SEVO (*n* = 271)	17.8 ± 2.9	NS	11.0 ± 0.6	<0.001	139.2 ± 15.6^a^	NS
	ISO (*n* = 281)	12.8 ± 3.1		16.4 ± 0.6		165.9 ± 16.3^a^	
Jindal et al. [2]	SEVO (*n* = 50)			6.8 ± 2.3	<0.05	193.2 ± 22.6^b^	NS
	DES (*n* = 50)			4.2 ± 1.5		188.4 ± 22.3^b^	
La Colla et al. [18]	SEVO (*n* = 14)	16.4 ± 1.5	<0.001	11.7 ± 2.2	<0.001	27 ± 1.6^a^	<0.001
	DES (*n* = 14)	9.4 ± 1		7.2 ± 1.8		16.3 ± 1.4^a^	
Saros et al. [19]	SEVO (*n* = 35)	5.3 ± 2.4	<0.05			140 ± 38^b^	NS
	DES (*n* = 35)	4.1 ± 2.0				143 ± 48^b^	
White et al. [20]	SEVO (*n* = 65)			8 ± 5	<0.05	90 ± 31^b^	NS
	DES (*n* = 65)			5 ± 3		98 ± 35^b^	
Magni et al. [153]	SEVO (*n* = 60)	15.2 ± 3	<0.0001	12.2 ± 4.9	NS		
	DES (*n* = 60)	11.3 ± 3.9		10.8 ± 7.2			

*DES* desflurane, *ISO* isoflurane, *LMA* laryngeal mask airway, *NS* not significant, *PRO* propofol, *SEVO* sevoflurane

^a^From PACU; ^b^ home

Studies in obese patients have compared emergence times between sevoflurane and desflurane (Table 3), which have the lowest fat solubility among the volatile anesthetics. The lower tissue and blood/gas solubility of desflurane accounts for the more rapid rate of recovery versus sevoflurane [22]. Strum et al. noted a nearly 9-min faster time to eye opening and a more than 11-min faster time to extubation with desflurane [23]. McKay et al. and Bilotta et al. also observed faster times to respond to commands and extubation with desflurane [24, 25]. However, Vallejo et al. suggested the difference in emergence time is minimal when the concentration of the inhaled agent is ≤80% of the maintenance dose at the time of discontinuation [26]. Similarly, Arain et al. demonstrated that potential differences in emergence time are not significant when proper titration is employed [22].

**Table 3:** Emergence times following anesthetic discontinuation (obese patients)

Study	Anesthetic agents compared	Extubation/LMA removal (min ± SD)	*P* value	Response to commands or eye opening (min ± SD)	*P* value
Strum et al. [23]	SEVO (*n* = 25)	25.5 ± 12	<0.0003	18.5 ± 8.7	<0.0001
	DES (*n* = 25)	14.2 ± 8		9.9 ± 4.5	
Bilotta et al. [24]	SEVO (*n* = 28)	15 ± 5	<0.001	12 ± 7	<0.001
	DES (*n* = 28)	7 ± 4		5 ± 3	
McKay et al. [25]	SEVO (*n* = 60)			6.6 ± 4.2	<0.001
	DES (*n* = 60)			4.0 ± 1.9	
Vallejo et al. [26]	SEVO (*n* = 30)	9.4 ± 5.9	NS	5.6 ± 4.1	NS
	DES (*n* = 34)	7.8 ± 5.1		4.6 ± 3.6	
Arain et al. [22]	SEVO (*n* = 20)	6.3 ± 0.8	NS	4.6 ± 0.7	NS
	DES (*n* = 19)	6.7 ± 0.7		5.1 ± 0.7	

*DES* desflurane, *LMA* laryngeal mask airway, *NS* not significant, *SEVO* sevoflurane

The most common adverse events associated with sevoflurane in studies of adult patients include nausea/vomiting, somnolence, coughing, shivering/chills, pain, dizziness, and confusion [17, 20]. Within the population of obese patients, the most commonly reported adverse events are nausea/vomiting and pain [23, 24, 26].

### Pediatric patients

Studies in pediatric populations have compared sevoflurane with desflurane [27–30], isoflurane [9, 27, 31], or propofol (Tables 1, 4) [6, 32, 33]. Sevoflurane has been described as the agent of choice for mask induction in children due to its lack of airway irritation, hemodynamic characteristics, and lower pungency [3]. In contrast, both desflurane and isoflurane are considered less suitable due to airway irritation [34].

**Table 4:** Emergence and discharge times following anesthetic discontinuation (pediatric patients)

Study	Anesthetic agents compared	Extubation/LMA removal (min ± SD)	*P* value	Eye opening (min ± SD)	*P* value	Discharge (min)	*P* value
Isik et al. [28]	SEVO (*n* = 40)	8.6	<0.05	8.6	<0.05		
	DES (*n* = 40)	6		5.6			
Kim et al. [29]	SEVO (*n* = 340)	9.3 ± 3.7	<0.001	9.2 ± 3.6	<0.001	35.5 ± 11.2^a^	0.001
	DES (*n* = 159)	6.2 ± 2.7		6.6 ± 3		32.2 ± 6.4^a^	
Ghoneim et al. [27]	SEVO (*n* = 20)	14.1 ± 0.79	<0.001 (SEVO/DES versus ISO)	11.7 ± 0.7	<0.001 (SEVO/DES versus ISO)		
	DES (*n* = 20)	11.6 ± 0.77		9.7 ± 0.5			
	ISO (*n* = 20)	21.25 ± 0.69		15.5 ± 0.9			
Jindal et al. [9]	SEVO (*n* = 42)	5.3 ± 2	<0.001	4.4 ± 2.7	<0.001		
	ISO (*n* = 42)	9.7 ± 4.2		8.1 ± 3.1			
Singh et al. [31]	SEVO (*n* = 40)	6.4 ± 3.3	<0.001	7.8 ± 3.4	<0.001	140.7 ± 49.3^a^	NS
	ISO (*n* = 40)	10.7 ± 4.6		12.8 ± 5.6		146 ± 43.3^a^	
Chandler et al. [6]	SEVO (*n* = 47)	21 ± 10	0.044			50 ± 16^a^	0.0004
	TIVA (*n* = 47)	25 ± 11				68 ± 28^a^	
Lopez Gil et al. [33]	SEVO (*n* = 60)	3.9 ± 1.7	<0.0001				
	PRO (*n* = 60)	5.8 ± 2.1					
Konig et al. [32]	SEVO (*n* = 91)					60 (42, 71)^b,c^	0.02
	PRO (*n* = 88)					70 (55, 85)^b,c^	

*DES* desflurane, *ISO* isoflurane, *LMA* laryngeal mask airway, *NS* not significant, *PRO* propofol, *SEVO* sevoflurane, *TIVA* total intravenous anesthesia

^a^From PACU; ^b^ median (25th, 75th percentiles); ^c^ home

In general, emergence and early recovery are faster in pediatric patients with desflurane than sevoflurane [28–30], though a recent study by Ghoneim et al. reported no difference between sevoflurane and desflurane, whereas emergence was longer with isoflurane [27]. Other studies have similarly found emergence from anesthesia to be longer with isoflurane than sevoflurane [9, 31]. The faster emergence of desflurane does not translate into a measurable discharge benefit [29, 30].

Relatively few studies have compared sevoflurane with propofol for induction of anesthesia in pediatric patients. Quality of induction, as characterized by intubation conditions [35] and behavior during induction [6], does not appear to differ between the agents. In contrast to findings in adult patients, one study reported a significantly more rapid induction with sevoflurane versus propofol. However, the authors noted that the dose of propofol used was relatively low (3 mg/kg), which may have been responsible for the discrepant results [33]. Consistent with studies in adult patients, measures of recovery from anesthesia (time to extubation [6, 33] and readiness for discharge [6, 32]) appear to favor sevoflurane compared with propofol.

Adverse events most commonly observed in pediatric patients during recovery from sevoflurane anesthesia include nausea/vomiting, coughing, shivering, pain, and agitation/delirium [27, 32, 33, 36, 37].

### Elderly patients

Age-related changes in elderly patients (>65 years of age) affect many organs, but of particular interest to anesthesiologists are the respiratory, cardiovascular, renal, and central nervous systems. These alterations have significant clinical implications for the different stages in anesthesia. This may be illustrated through the inverse relationship between age and minimal alveolar concentration (MAC) that has been observed for halogenated inhaled anesthetics, in which elderly patients require a lower anesthetic MAC to achieve the same effectiveness as adults or children. The MAC for sevoflurane in elderly patients is 1.48%, which is lower than the MAC for children (2.49%) and adults (1.71–2.05%) [38, 39].

Yamaguchi et al. demonstrated that sevoflurane induction is well tolerated [40]. Peduto et al. investigated the recovery from sevoflurane-induced anesthesia in elderly patients compared with isoflurane (Table 5) [41]. The time to extubation, emergence, response to command, and suitability for discharge were shorter for sevoflurane, leading the authors to conclude that sevoflurane provides a more rapid emergence than isoflurane. In a similar comparison of sevoflurane versus desflurane anesthesia in elderly patients, the recovery time was reported to be faster with desflurane [42]. However, the discharge time from the postanesthesia care unit was similar for patients who received desflurane and those who received sevoflurane, even with the previously noted difference in early recovery [42, 43].

**Table 5:** Emergence and discharge times following anesthetic discontinuation (elderly patients)

Study	Anesthetic agents compared	Extubation/LMA removal (min)	*P* value	Eye opening (min)	*P* value	Discharge from PACU (min)	*P* value
Peduto et al. [41]	SEVO (*n* = 50)	8 (2–35)^a^	<0.01	8.5 (2–57)^a^	<0.01	21 (5–69)^a^	<0.01
	ISO (*n* = 54)	11 (2–35)^a^		12.5 (3–47)^a^		27.5 (9–180)^a^	
Heavner et al. [42]	SEVO (*n* = 25)	9 (5–13)^b^	<0.05	11 (8–16)^b^	<0.05	71 (61–81)^b^	NS
	DES (*n* = 25)	5 (4–9)^b^		5 (3–5)^b^		56 (35–81)^b^	
Pakpirom et al. [43]	SEVO (*n* = 38)	12.4 ± 5.5	NS	9.6 ± 4.6	0.04	49.4 ± 23.1	NS
	DES (*n* = 42)	10.4 ± 3.7		7.5 ± 3.4		50.1 ± 25.8	

*DES*  desflurane,* ISO* isoflurane,* LMA* laryngeal mask airway,* NS* not significant,* SEVO* sevoflurane

^a^Median (range)

^b^Median (interquartile range)

A recent meta-analysis of randomized controlled trials in elderly patients showed that recovery times were shorter for desflurane than for sevoflurane. The meta-analysis also addressed cognitive dysfunction and delirium upon emergence from anesthesia and revealed no difference in the incidence of cognitive dysfunction between these agents [7]. This indicates that the shorter recovery time does not translate into a significant effect on cognition; other factors play more important roles.

The most commonly reported adverse events associated with sevoflurane following surgery in elderly include nausea/vomiting, pain, dizziness [42, 44], delirium [45], and short-term cognitive dysfunction [11, 45, 46].

### Additional safety considerations

As with other inhaled anesthetics in this class (e.g., isoflurane and desflurane), sevoflurane affects a variety of systems during anesthesia that can result in cardiovascular (hypotension and cardiac depression), central nervous system (CNS) (depressed function), and respiratory (ventilatory depression) side effects, which are well described in the literature [39]. Postoperative adverse events associated with inhaled anesthetics include pain, nausea/vomiting, headache, dizziness, cognitive dysfunction, and delirium [39]. Postoperative nausea/vomiting (PONV) is one of the most frequent adverse events, occurring in approximately 33% of patients. This pro-emetic effect is more pronounced in the early postoperative period (0–2 h postsurgery), and does not appear to differ among the various volatile anesthetics [47]. This increase in PONV has also been observed in pediatric patients [30, 32, 37]. PONV is of significant concern to patients; however, PONV can be successfully managed through anesthesia modification (e.g., use of air rather than N_2_O) and/or antiemetic administration [48]. It was recently noted in a meta-analysis that a volatile gas plus one anti-emetic drug is comparable to propofol in terms of rate of PONV [49]. The same meta-analysis also showed that patients receiving TIVA had a significantly higher risk of experiencing PONV in the late postoperative phase (RR 1.41, 95% CI 1.10, 1.79, *P* = 0.006). Based on these data, it is in the hands of the anesthesiologist to decide which therapeutic approach is most suitable for an individual patient. Other uncommon yet important adverse events identified with use of sevoflurane include hyperkalemia [50], seizures [51, 52], hepatic dysfunction [53, 54], and malignant hyperthermia [55].

In summary, numerous investigations demonstrate the efficacy and safety of sevoflurane for both induction and maintenance of general anesthesia in pediatric, adult, obese, and elderly patient populations. Across the patient populations, evidence suggests that emergence times from sevoflurane anesthesia are generally faster than from isoflurane anesthesia and slower than from desflurane anesthesia, whereas there appears to be no difference in readiness for discharge. Comparisons between sevoflurane and propofol have also shown fairly consistent results; more rapid induction has been noted with propofol and faster emergence and readiness for discharge has been observed with sevoflurane. In addition, the safety profile of sevoflurane has been established in these populations. The most common side effects of sevoflurane are similar to those observed with other inhaled anesthetics.

## Emerging topics in inhalation anesthesia

### Hemodynamic stability and organ protection

#### Hemodynamics

Volatile anesthetics have an impact on the cardiovascular system, either through effects on the myocardium itself or by decreasing systemic vascular resistance [56, 57]. Clinically, changes in heart rate and blood pressure are most important because, in patients at an increased risk of cardiovascular complications, both hypotension and tachycardia may cause a dramatic increase in the incidence of perioperative adverse events [58, 59].

Studies in human volunteers and patients have not shown an increase in heart rate with clinically used concentrations of sevoflurane [60]. The incidence of arrhythmias has been studied in patients undergoing cardiac surgery. In a retrospective analysis of 460 patients who were anesthetized with sevoflurane, desflurane, propofol, or midazolam, Cromheecke et al. found a significantly lower incidence of atrial fibrillation during the first 24 h following cardiopulmonary bypass (CPB) only with sevoflurane (6% versus 21, 15, and 17%, respectively; *P* = 0.004) [61]. These results were confirmed and extended for the incidence of both atrial fibrillation and supraventricular tachycardia in a more recent investigation of patients during off-pump cardiac surgery during which continuous ECG monitoring was utilized for up to 72 h after surgery [62].

Cardiac output and both coronary and cerebral blood flow have been the foci of several studies. Although sevoflurane decreased myocardial contractility in dogs by approximately 40% in a dose-dependent fashion, as assessed by preload recruitable stroke work [63], these findings could not be confirmed in human volunteers at sevoflurane concentrations ranging from 0 to 2 MAC [64]. Taking the currently available evidence together, cardiac output at clinically relevant concentrations is not substantially decreased, but is usually preserved [65, 66]. In addition, there is also no evidence for a relevant coronary steal phenomenon in patients during sevoflurane anesthesia.

Sevoflurane acts as a weak coronary vasodilator [67], with coronary blood flow maintained. Also, as in other vascular beds, it acts as a cerebral vasodilator [3]. Although early papers suggested a preserved cerebral autoregulation with sevoflurane up to 1.5 MAC in humans [68, 69], recent investigations reported a shortened autoregulatory plateau range [70] and an impaired autoregulatory capacity at higher concentrations (i.e., at BIS values of 35 and sevoflurane concentrations ranging between 2 and 4%) [71]. Although the clinical impact of these data is unclear, these findings should be kept in mind in order to adequately adjust mean arterial pressure in the setting of CPB surgery [72].

### Cardiac protection

In a pivotal paper, Kersten et al. showed that the protective effect of isoflurane involved the activation of potassium-activated ATP (K_ATP_) channels, a phenomenon known as anesthetic preconditioning [73]. Shortly thereafter, Preckel et al. reported beneficial effects of enflurane, isoflurane, sevoflurane, and desflurane on reperfusion injury following myocardial ischemia in rabbits [74]. Two groups of researchers independently translated these findings into daily clinical practice [75, 76]. They chose cardiac surgery as a model because a reproducible and well-defined episode of myocardial ischemia is part of the surgical procedure in cardiac surgery. Numerous subsequent experimental and clinical studies have addressed the concept of anesthetic-induced cardioprotection. For reasons discussed above, most clinical studies have been performed in the field of cardiac surgery, either with or without CPB. Although the experimental evidence for anesthetic preconditioning and postconditioning is unequivocal and indisputable, the significance of anesthetic-induced cardioprotection in the clinical setting is still controversial.

Several laboratory investigations have revealed that ischemic preconditioning and anesthetic-induced preconditioning share a common phenotype. Based on current evidence, two main intracellular signal transduction pathways are thought to convey cardioprotection by transducing signals from cellular receptors to ion channels located in the mitochondria. Briefly, both the reperfusion injury salvage kinases pathway, via G-protein-coupled receptors, and the survivor-activating factor enhancement pathway are most likely involved [77]. The final pathway is the inhibition of the opening of the mitochondrial permeability transition pore (mPTP) and facilitated opening of K_ATP_ channels. Protective actions of sevoflurane on endothelial cells have also been shown in human volunteers, which may yield additional beneficial effects [78]. Finally, sevoflurane administration may reduce inflammatory markers. In a recent meta-analysis of patients undergoing coronary artery bypass grafting (CABG) surgery, sevoflurane pretreatment significantly reduced concentrations of the pro-inflammatory cytokines interleukin (IL)-6 and IL-8 compared with controls [79].

After publication of the first promising results from clinical studies, the issue of anesthetic-induced preconditioning gained a great deal of interest. De Hert and others demonstrated that sevoflurane reduced troponin I release following cardiac surgery with CPB and after aortic valve replacement or mitral valve surgery [80–83]. In one of these studies, which compared sevoflurane with propofol, the greatest effect on troponin release was observed when sevoflurane was applied before, during, and after CPB.

In the largest trial published thus far, enrolling 414 patients, De Hert et al. observed a significant difference between total intravenous anesthesia (TIVA) and sevoflurane in 1-year mortality (12.3 versus 3.3%, respectively; *P* = 0.034) [84]. Also, with respect to long-term outcome, the incidence of late cardiac events 1 year after cardiac surgery with CPB was significantly less in a randomized, controlled, double-blind study of patients who received 2 MAC sevoflurane or placebo (3 versus 17%; *P* = 0.038) on CPB for 10 min before aortic cross-clamping [85]. In addition, evidence from retrospective “real-life” database analyses of patients undergoing CABG surgery in Italy (*N* = 34,310 patients) and in Denmark (*N* = 10,535 patients) suggested that the use of volatile anesthetics is associated with a reduced 30-day mortality [86, 87]. Bignami et al. reported significantly reduced 30-day mortality rates with volatile anesthetics (*P* = 0.035) and with increased duration of volatile anesthetic use during surgery (*P* = 0.022) [86], whereas Jakobsen et al. found significant reductions in 30-day mortality with sevoflurane versus propofol in patients without a previous myocardial infarction (2.49 versus 3.55%, *P* = 0.025) or without unstable angina (2.28 versus 3.14%, *P* = 0.015) [87].

Despite this relatively robust evidence and American College of Cardiology Foundation/American Heart Association guidelines recommending the use of inhaled anesthetics in patients undergoing cardiac surgery with CPB (class IIa, evidence level A) [88], there are still doubts among clinicians about the efficacy of anesthetic-induced cardioprotection in daily practice. These doubts have been fueled by several trials that failed to show beneficial effects of sevoflurane in cardiac or major vascular surgery. A French dual-center study failed to demonstrate a significant cardioprotective effect after 15 min of sevoflurane administration prior to CPB [89]. In the most recent randomized study, enrolling 200 patients, Landoni et al. did not find a difference between patients anesthetized with sevoflurane or propofol in postoperative cardiac troponin release, 1-year all-cause mortality, rehospitalizations, and adverse cardiac events [90]. In major vascular surgery, Lurati-Buse et al., Lindholm et al., and Zangrillo et al. all failed to show beneficial effects of a sevoflurane-based anesthesia regimen compared with TIVA [91–93].

In the most recent meta-analysis of sevoflurane-induced cardioprotection in patients undergoing cardiac surgery, which included 15 studies with a total of 1646 patients, the authors found improved postoperative cardiac output, reduced postoperative 24-h cardiac troponin I concentrations, reduced postoperative usage of inotropes and pressors, a shorter ICU stay, and a decreased incidence of atrial fibrillation [94]; mortality and major morbidity, however, were not different between groups.

### Neuronal effects of sevoflurane

Pharmacologic neuroprotection from cerebral ischemia and reperfusion continues to be an area of interest. Animal studies have suggested that ischemic neuronal injury results from early excitotoxic cell death mediated by excessive release of glutamate, and by oxidative stress caused by reperfusion injury together with delayed cell death as a result of apoptosis [95, 96]. However, early neuroprotective effects of volatile anesthetics in animal models indicate a beneficial effect in reducing excitotoxicity and only minimal effects on delayed cell death caused by apoptosis. Isoflurane has been demonstrated to reduce excitotoxic cell death in vitro [97] and in vivo [98] in models of focal [99, 100], hemispheric [101], and global [102, 103] ischemia. Desflurane and halothane were observed to have neuroprotective properties if used at 1.5 MAC during the ischemic event when compared with awake rats subjected to an ischemic insult [102].

Sevoflurane has shown anesthetic preconditioning (APC) potential [104]. Wang et al. reported that repeated sevoflurane APC reduced infarct size in rats after focal ischemia, and further investigated whether the inhibition of apoptotic signaling cascades contributes to sevoflurane APC-induced neuroprotection. Male rats were exposed to ambient air or 2.4% sevoflurane for 30 min per day for four consecutive days and then subjected to occlusion of the middle cerebral artery for 60 min at 24 h after the last sevoflurane intervention. APC with sevoflurane markedly decreased apoptotic cell death, which suggests that suppression of apoptotic cell death contributes to the neuroprotection against ischemic brain injury [105].

Despite some evidence of neuroprotective effects of inhaled anesthetics, conflicting data have also been reported. Extensive studies in neonate rodents have shown that postnatal exposure to high doses of commonly used anesthetic drugs, in isolation or in combination for a period of several hours, can induce apoptotic cell death with potentially long-term functional consequences [106]. Postexposure environmental factors also appear to play a substantial role in the expression of neurotoxicity [107]. General anesthetics can be a potential cause of neurologic sequelae in animal models, such as deficits in learning and memory [108, 109]. In addition, it should be noted that animal studies demonstrating neuroprotection by inhaled anesthetics have failed to translate to the clinical trial setting and, to date, it remains unclear if a quantitative and qualitative correlation can be made between the effects on rodents and those in humans. Therefore, despite research efforts, clinical evidence for ischemic brain protection by inhaled anesthetics remains controversial [110].

In summary, evidence demonstrates cardioprotective effects of inhaled anesthesia during cardiac surgery and, despite some contradictory findings, it is conceivable that sevoflurane also exerts cardioprotective effects outside cardiac surgery. The potentially beneficial effects may be diminished or even abolished by advanced age, diabetes, and myocardial remodeling. Therefore, it is of paramount importance to tailor the cardioprotective technique to the individual patient (e.g., by enhancing stimulus intensity through repeated sevoflurane wash-in and washout) and to avoid the use of drugs that are capable of abolishing cardioprotection in patients at risk for perioperative cardiovascular complications. Preliminary evidence also suggests that sevoflurane may possess neuroprotective properties in hypoxia/ischemia; however, a substantial amount of research is necessary to confirm the neuroprotective effect in the clinic.

### Behavioral outcomes associated with inhaled anesthesia: postoperative cognitive dysfunction and postoperative delirium

#### Adult and elderly patients

Postoperative cognitive disturbances may take various forms, including POCD and POD [111, 112]. Cognitive recovery after anesthesia is multifactorial and depends on the type of anesthesia used, the type of surgery, and individual patient characteristics such as age [113]. For example, the incidence of POCD after cardiac surgery has been found to range between 50 to 70% in the first postoperative week, 30–50% after 6 weeks, and 20–40% at 6 months and 1 year [114].

Postoperative deficits in cognitive function were observed more than 60 years ago by Bedford [115]. Recent studies have consistently revealed a high incidence of POCD in older patients, though with a wide range of results [116–121]. Investigations of small patient populations have revealed POCD incidences of 56% at 3 months (*N* = 140) [116], 44.8% at 6–12 weeks (*N* = 29) [117], and 18% at 3 months (*N* = 37) [121]. Two studies are notable due to their large patient populations. The ISPOCD study found a significantly increased incidence of POCD in elderly patients undergoing major noncardiac surgery (*N* = 1218) compared with healthy controls (*N* = 176) at both 1 week and 3 months after surgery, with elderly patients exhibiting POCD incidence rates of 25.8 and 9.9% at these time points, respectively [119]. These findings were supported in a study using a similar methodology, as Monk et al. also reported an increased incidence of cognitive dysfunction at hospital discharge in adults of all ages (ranging from 30.4 to 41.4%), but younger adults did not have an increased risk for POCD [120, 122].

A meta-analysis evaluating POCD in relation to the anesthetic technique (regional versus general anesthesia) in patients undergoing noncardiac surgery was conducted by Guay et al. Twenty-six RCTs including 2365 patients, 1169 for regional anesthesia and 1196 for general anesthesia, were examined. The standardized difference in means for the tests included in the 26 RCTs was not significant. The analysis was blinded to the anesthetic technique for 12 of the RCTs, including 798 patients. The authors concluded that the meta-analysis did not support the concerns that a single exposure to general anesthesia in an adult can significantly contribute to permanent POCD after noncardiac surgery [123].

Data on the incidence and severity of short-term cognitive dysfunction (i.e., transient deficits of only a few days) and POD relating to specific inhalation anesthetic agents are unclear. Rortgen et al. found no difference in the incidence of cognitive dysfunction between sevoflurane and desflurane [11], and a recent meta-analysis revealed no difference in short-term cognitive dysfunction between elderly patients undergoing sevoflurane or desflurane anesthesia [7]. Both sevoflurane and desflurane significantly decreased Mini-Mental State Examination (MMSE) scores 1 h after surgery, an effect observed in 51–57% of the patients. These cognitive deficits are short-lived, as the MMSE scores returned to normal for both agents 3–6 h after surgery [44]. Similar results have been reported for a study comparing sevoflurane with isoflurane [124]. It should be noted, though, that a recent study by Tachibana et al. revealed a significantly greater impairment in MMSE scores with sevoflurane than desflurane at 24 h postsurgery [12].

Few studies in elderly patients have addressed potential differences between sevoflurane and propofol in behavioral outcomes, and a consistent picture is yet to emerge. One study, which compared the effects of sevoflurane with propofol on the incidence of delirium in elderly patients, found that sevoflurane-treated patients did not exhibit POD based on the Delirium Rating Scale (DRS), and the authors concluded that sevoflurane is preferable to propofol [45]. However, in elderly patients with pre-existing mild cognitive impairment, sevoflurane has been found to have a more severe effect on cognitive function 7 days after surgery compared with propofol [46], and at 2 years postsurgery [125]. Conflicting data have also been reported between sevoflurane and propofol in adult patients. Both Schoen et al. and Goswami et al. have found greater short-term cognitive dysfunction with propofol than sevoflurane [8, 126], whereas Kalimeris et al. showed the opposite [127]. It is clear that additional research is required to elucidate any differences between sevoflurane and propofol in behavioral outcomes in adults and the elderly.

#### Pediatric patients

Anesthesia in the pediatric population represents an area of specific concern for behavioral outcomes. Recent studies in animal models have suggested the potential for long-term neurodevelopmental deficits following early exposure to anesthesia. One study in rhesus monkeys demonstrated significant neuronal damage following combined anesthesia with isoflurane and N_2_O [128]. However, observational studies in children have produced mixed results, and there have been few investigations of specific anesthetic agents and their potential neurodevelopmental effects. A group of studies from the Mayo Clinic has shown that multiple anesthetic exposures in infants and children, but not a single exposure, increase the risk of learning disabilities and later development of attention deficit hyperactivity disorder (ADHD) [129–131]. Other studies have demonstrated that a single exposure to general anesthesia causes increased risk of developmental disorders and deficits in language/abstract reasoning in children <3 years of age [132–134]. Still other reports have found no association between exposure of children to general anesthesia and the development of abnormal behavior or poor academic performance later in life [135–137].

It has been noted in a recent editorial by Rappaport et al. that there is a substantial need for additional human data in this area due to the paucity of prospective controlled trials [138]. To this end, two such studies have been published within the last year. A recent randomized controlled study has reported a difference in negative behavioral changes (NBCs) between inhaled anesthesia (sevoflurane and N_2_O) and TIVA [139]. Pediatric patients undergoing adenotonsillectomy were assessed with the Post-Hospitalization Behavior Questionnaire up to 6 months postsurgery. The results indicated that patients in the inhaled anesthesia group had an increase in NBCs (primarily anxiety and withdrawal) compared with TIVA, which was associated with an absolute risk reduction of 0.55 for at least one NBC at the 6-month time point. Of potentially greater significance, Davidson et al. recently reported secondary outcomes from a study of more than 700 enrolled infants who were randomly assigned to receive awake-regional versus sevoflurane general anesthesia (median sevoflurane exposure = 54 min). At 2 years of age, mean cognitive composite scores were similar in both groups (awake-regional = 98.6 ± 14.2; sevoflurane = 98.2 ± 14.7), leading the authors to conclude that sevoflurane exposure of less than 1 h during infancy does not increase the risk of adverse neurodevelopmental outcomes. The primary outcome of this trial is IQ score at 5 years of age, which will yield further clarity on this important issue [140].

Inhalational anesthetics have been associated with POD in children, which manifests as a variety of behavioral disturbances, including confusion, irritability, and disorientation. Estimates indicate rates of POD range from 10 to 67% of pediatric anesthesia cases [141–143]. There is little evidence to suggest the existence of significant differences among inhalational anesthetics in pediatric POD. Locatelli et al. reported similar incidence rates of POD for sevoflurane (25%) and desflurane (25%) in the pediatric population [144], and comparable results have been found in other studies [9, 145, 146]. Conflicting data have been reported in comparisons between sevoflurane and propofol. A recent randomized trial in 112 children 2–6 years of age found a significantly greater incidence of POD with sevoflurane maintenance than with propofol maintenance (38.3 versus 14.9%, respectively; *P* = 0.018) [6]. Picard et al. reported similar findings, though the increased incidence of POD with sevoflurane did not result in a longer time to discharge [142]. In contrast, Konig et al. reported no difference between sevoflurane and propofol in the incidence of POD in pediatric patients undergoing ambulatory dental surgery [32].

### Novel research in anesthesia: optimizing emergence from general anesthesia

Upon discontinuation of general anesthesia, patients typically remain unconscious for several minutes and then progress through a series of stages concluding with full consciousness. In general, the stages of emergence include the return of spontaneous respiration, patient behaviors prompting extubation, eye opening, and the ability to respond to questions [147]. Recently, novel approaches aimed at accelerating emergence and improving patient outcomes have been proposed and are under investigation.

CNS stimulants, which promote wakefulness and exhibit pro-cognitive effects in humans, may be useful for speeding up the emergence process. These drugs include amphetamine and methylphenidate, which are approved for the treatment of ADHD, and modafinil and armodafinil, which are approved for the treatment of narcolepsy. Animal studies have revealed decreased emergence times with methylphenidate [148] and dopamine receptor agonists [149] in rats anesthetized with isoflurane. A clinical study of methylphenidate investigating its effects on emergence time and short-term cognitive dysfunction is underway [150]. Demonstrating accelerated emergence and improved cognitive function with methylphenidate could provide physicians with an important new tool for the postanesthesia care of patients.

Beyond reducing the time to emergence from anesthesia, the potential to ameliorate both POD and POCD following surgery is of significant medical interest. A short-term treatment with pro-cognitive agents could potentially reduce the incidence of POCD. As mentioned, methylphenidate is being studied in this regard. In addition, amantadine, a dopamine agonist/weak NMDA antagonist, has been found in an animal model to reduce cognitive impairment following propofol anesthesia [151]. It is interesting to note that memantine, an NMDA antagonist, is approved for the treatment of dementia in Alzheimer’s disease. Consequently, it is tempting to speculate that memantine or other treatments for Alzheimer’s disease such as donepezil or rivastigmine, which exert their therapeutic effects through the inhibition of acetylcholinesterase, may be beneficial too. The potential also exists for the use of nonpharmacologic methods in the prevention of POCD. A recent study by Saleh et al. randomized elderly patients undergoing surgery to receive preoperative cognitive training during three 1-h sessions or no cognitive intervention. Neuropsychological testing 1 week after surgery revealed a significantly reduced incidence of POCD in the intervention group (15.9%) compared with the control group (36.1%) [152].

## Summary and conclusions

A wide variety of investigations have demonstrated the efficacy and safety of sevoflurane for both induction and maintenance of general anesthesia across a broad range of patient populations. Indeed, induction of anesthesia with sevoflurane is favorable in pediatric patients in comparison with isoflurane and desflurane due to its lack of airway irritation. Comparisons of general anesthetics in emergence from anesthesia have shown that emergence times from sevoflurane are generally faster than from isoflurane. Emergence from sevoflurane is slower than from desflurane, with no difference in readiness for discharge. However, sevoflurane is faster in both emergence and readiness for discharge than propofol. These differences between sevoflurane and propofol are small and may not be clinically relevant; however, a more rapid emergence and readiness for discharge may be beneficial in the ambulatory surgery setting.

Clinically, the smaller impact of sevoflurane on hemodynamic and cardiovascular parameters is favorable when considering the association of heart rate and blood pressure with perioperative adverse events. Sevoflurane does not increase heart rate at clinically used concentrations, whereas isoflurane and desflurane may cause a marked increase following a rapid increase in inspiratory concentration; conversely, propofol may cause bradycardia. Moreover, this cardiac stability appears to extend to the postoperative period (24–72 h after surgery), which may convey additional benefits for reducing morbidity and mortality [60, 62, 94]. Clinical studies examining the relationship between hemodynamic/cardiac parameters and patient outcomes are needed to further clarify any potential benefits of these characteristics.

In recent years, the cardioprotective properties of sevoflurane have been demonstrated in patients undergoing cardiac surgery, but the potential beneficial clinical effects of sevoflurane outside cardiac surgery are still under debate. In addition, despite the relatively robust evidence on reduced 30-day and 1-year mortality, and the recommended use of inhaled anesthetics in patients undergoing cardiac surgery with CPB, there are still doubts among clinicians about the efficacy of anesthetic-induced cardioprotection in daily practice. Clinical research should focus on specific preconditioning algorithms that are able to fully exploit the inherent cardioprotective properties of sevoflurane, as well as the implementation of these algorithms in clinical practice.

Cognitive function is impaired after surgery as patients experience deficits in attention and in short-term and long-term memory, as indicated by the prevalence of POD and POCD in the majority of studies conducted in this field. POD is observed shortly after surgery, while data on POCD ranges from 1 week to 1 year after surgery in adults/elderly. With regards to the pediatric population, the direct effect of anesthesia on neuronal development adds another complex variable. It is clear that there is an association between general anesthesia and POD/POCD; however, no conclusions can be drawn about which anesthetic agents may be more or less likely to precipitate them. It is difficult to distinguish between the effects of anesthesia on the brain versus surgery-related effects such as inflammation. Well-designed prospective studies investigating the specific long-term effects of the various inhalational anesthetic agents are needed in order to reach definitive conclusions.

The practice of anesthesia continues to advance through research into ways in which postanesthesia outcomes may be improved. It could be hypothesized that the use of pro-cognitive agents to accelerate emergence from anesthesia could enable the rapid discharge of patients and potentially improve patients’ cognitive skills at the time of discharge. An enhanced psychological outcome of patients after anesthesia is an attractive notion in view of the association of general anesthesia with adverse behavioral outcomes. Inhaled anesthetics have been linked to cognitive deficits, and there is no evidence that any particular anesthetic is related to POD/POCD to a greater or lesser degree than any other.

Strategies for improving neuropsychological outcomes should not be limited to pharmacological interventions. Nonpharmacological interventions, such as perioperative hypothermia or cognitive training, may be beneficial. However, baseline measures are needed to properly assess the benefit of such interventions.

In conclusion, over the past 20 years, sevoflurane has been used in nearly 900 million patients and has demonstrated a positive benefit–risk ratio over a broad spectrum of patients. Moreover, the use of pro-cognitive agents to minimize the cognitive deficits of anesthesia and the creation of new guidelines to improve cardiovascular management as well as neuropsychiatric outcomes may ultimately improve the safety of anesthesia.
